# Dynamic changes in marital status and survival in women with breast cancer: a population-based study

**DOI:** 10.1038/s41598-021-84996-y

**Published:** 2021-03-08

**Authors:** Wu Ding, Guodong Ruan, Yingli Lin, Jianming Zhu, Chuanjian Tu, Zhian Li

**Affiliations:** 1 Department of Oncological Surgery, Shaoxing Second Hospital, Shaoxing, China; 2 Department of Neurosurgery, Shaoxing Central Hospital, Shaoxing, China; 3 Department of Clinical Medicine, Shaoxing University School of Medicine, Shaoxing, China; 4 Department of Early Childhood Education, Shaoxing Vocational and Technical College, Shaoxing, China

**Keywords:** Breast cancer, Disease-free survival

## Abstract

Marital status proved to be an independent prognostic factor for survival in patients with breast cancer. We therefore strove to explore the impact of dynamic changes in marital status on the prognosis of breast cancer patients. We selected patients meeting the eligibility criteria from the Surveillance, Epidemiology, and End Results cancer database. We then used multivariate Cox proportional hazard regression model to analyze the effect of dynamic changes in marital status on the prognosis of overall survival (OS) and breast cancer-specific special survival (BCSS). Compared with the patients in the Single–Single group and the divorced/separated/widowed–divorced/separated/widowed (DSW–DSW) group, patients in the Married–Married group were significantly associated with better BCSS (HR 1.13, 95% CI: 1.03–1.19, *P* < 0.001; HR 1.19, 95% CI: 1.14–1.25, *P* < 0.001, respectively) and OS (HR 1.25, 95% CI: 1.20–1.30, *P* < 0.001; HR 1.49, 95% CI: 1.45–1.54, *P* < 0.001, respectively). In contrast to the DSW–DSW group, the Single–Single group and the DSW–Married group showed similar BCSS (HR 0.98, 95% CI: 0.92–1.05, *P* = 0.660; HR 1.06, 95% CI: 0.97–1.15, *P* = 0.193, respectively) but better OS (HR 1.14, 95% CI: 1.09–1.19, *P* < 0.001; HR 1.32, 95% CI: 1.25–1.40, *P* < 0.001, respectively). Compared with the Single–Single group, the Single–Married group showed significantly better BCSS (HR 1.21, 95% CI: 1.07–1.36, *P* = 0.003) but no difference in OS (HR 1.08, 95% CI: 0.98–1.18, *P* = 0.102); In contrast to the Married–DSW group, the Married–Married group exhibited better BCSS (HR 1.11, 95% CI: 1.05–1.18, *P* < 0.001) and OS (HR 1.27, 95% CI: 1.22–1.32, *P* < 0.001). Our study demonstrated that, regardless of their previous marital status, married patients had a better prognosis than unmarried patients. Moreover, single patients obtained better survival outcomes than DSW patients. Therefore, it is necessary to proactively provide single and DSW individuals with appropriate social and psychological support that would benefit them.

## Introduction

Breast cancer is the most frequently diagnosed cancer and the second leading cause of cancer-related deaths in women in the Western world^1^. As a systemic disease, the formation of breast cancer is the result of complex interactions between physiological and psychosocial factors^2^. Marital status, as one of the most important forms of social relations, influences those factors of breast cancer patients. A growing body of evidence suggests that marital status acts as an independent prognostic factor for survival in patients who experience breast cancer, and the mortality of breast cancer is higher in unmarried patients^3–9^.

Previous studies have confirmed that married patients can get more mental and financial support, have better compliance, which can help them to be diagnosed at an early stage and get more appropriate treatments, ultimately prolonging their survival^10–13^. Nevertheless, those researches provide information on marital status only from the time points of recently diagnosed breast cancer which may not reflect the dynamic changes in long-term survival populations or account for marital status changes before or after the cancer diagnosis. Moreover, further analysis of marital status changes during the period of breast cancer, which may reveal the potential mechanism of marital status affecting the prognosis of breast cancer, is also neglected.

The Surveillance, Epidemiology, and End Results (SEER) cancer database includes research data from 18 different population-based cancer registries which have been widely used for this kind of study that explores the relation between marital status and breast cancer survival rates among patients^3,7–9,14^. In view of the current research situation, we selected the data from SEER database and conducted this analysis to explore the relationship between dynamic changes in marital status and overall survival (OS) or breast cancer-specific survival (BCSS).

## Materials and methods

### Patient selection and data extraction

**Figure 1:**
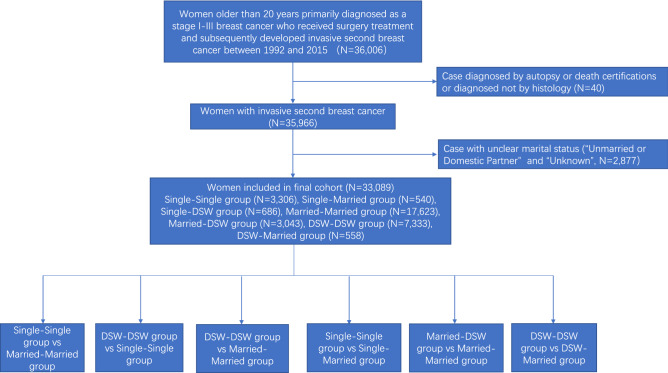
Flow diagram of patient population.

To estimate the correlation between the dynamic changes in marital status and BCSS or OS in breast cancer patients, we used SEER*Stat 8.3.6 software to extract relevant information, including patient identification, year of diagnosis, age at diagnosis, race/ethnicity, marital status, insurance, 6th tumour-node-metastasis (TNM) staging classification, histology type, nuclear grade, oestrogen receptor (ER) status, progesterone receptor (PR) status, surgery, chemotherapy, radiation therapy, cause-specific death classification, other cause of death classification, interval between primary and second breast cancer and survival month. We identified 36,006 patients over 20 years old who were first diagnosed with stage I–III primary breast cancer (BC) and received surgical treatment and subsequently developed invasive second primary breast cancer (SBC) from 1992 to 2015 (Fig. 1). The patients might have had different marital statuses when they were first and second diagnosed with breast cancer. Therefore, we defined a change in the marital status of patients with two breast cancer diagnoses as a dynamic change in the marital status of these patients. We excluded patients only diagnosed by autopsy or death certifications, without histological confirmation, or with marital status defined as “Unmarried or Domestic Partner” or “Unknown”. Eventually, 33,089 eligible BC patients were included in this study. Marital status at diagnosis was the primary variable, and the participants were classified as married, single, and divorced/separated/widowed (DSW). Therefore, according to the marital status of the primary BC and the SBC, participants were divided into seven groups: Single–Single group (N = 3306), Single–Married group (N = 540), Single–DSW group (N = 686), Married–Married group (N = 17,623), Married–DSW group (N = 3043), DSW–DSW group (N = 7333), DSW–Married group (N = 558). This study was reviewed and approved by the institutional review board at the Shaoxing Second Hospital, and the data were deidentified; thus, patient consent was not involved.

### Statistical analysis

We adopted statistical analytic approaches similar to those used in previous studies^15,16^ that examined the benefit of interventions for BC subsets. Differences in the patient characteristics of SBC were compared between every two groups using Pearson chi-square and T tests for categorical and continuous variables, respectively. Multiple imputation of missing data was performed by a multivariate logistic regression model, and 10 cycles were repeated to produce a final data set. The imputation model included the following variables: patient age (continuous), year of diagnosis, histology type (IDC, ILC and other), T stage, N stage, M stage, and chemotherapy (yes or no).

The inverse probability propensity score weighting^17^ was used to balance clinicopathological characteristics between every two groups, and we calculated the propensity scores based on patient age, race, year of diagnosis, insurance, histology type, nuclear grade, T stage, N stage, M stage, ER status, PR status, surgery, chemotherapy and radiotherapy by a logistic regression model for different marital statuses. Patient characteristics after propensity score adjustment are shown to be balanced in Appendix Tables 1–6 in the Supplement. Then, propensity score-weighted log-rank tests and Cox proportional hazards models were conducted to compare OS and BCSS between every two groups, and hazard ratios (HRs) with 95% confidence intervals (CIs) of BCSS (OS) were calculated from multivariable models that adjusted for year of diagnosis, age, race, year of diagnosis, insurance, histology type, nuclear grade, T stage, N stage, M stage, ER status, PR status, surgery, chemotherapy and radiotherapy. Similar procedures were also performed among subgroups with different ages, and interaction tests were conducted by using a likelihood ratio test to explore whether the survival benefit conferred by marital status varied across subgroups.

To assess the consistency of our findings, we conducted four types of sensitivity analyses. First, we repeated analyses after excluding the missing data containing insurance, ER, and PR status. Then, a proportional subdistribution hazards model was used to calculate the HR of BCSS between every two groups after adjusting for competing events such as death from other causes^18^. Third, the entire analyses were repeated after multiple imputation of unknown data by using random survival forest methodology.

All *P* values were calculated from 2-sided tests with a threshold of 0.05 to evaluate the statistical significance of the survival benefit by surgery, and all statistical analyses were performed using R software (version 3.6.1).

### Ethical approval

All procedures performed in studies involving human participants were in accordance with the ethical standards of the institutional and/or national research committee and with the 1964 Helsinki declaration and its later amendments or comparable ethical standards. For this type of retrospective study, formal consent is not required. The ethics committee of the Shaoxing Second hospital approved the study.

## Results

### Patients' baseline characteristics

In view of the inclusion criteria, this study included 33,089 patients diagnosed with SBC from 1992 to 2015. A flowchart of the study participant selection process is shown in Fig. 1. A summary of the baseline features of the patients in the seven marital status groups is shown in Table 1. Among eligible patients, 3306 patients were grouped into the Single–Single group, 540 patients were grouped into the Single–Married group, 686 patients were grouped into the Single–DSW group, 17,623 patients were grouped into the Married–Married group, 3043 patients were grouped into the Married–DSW group, 7333 patients were grouped into the DSW–DSW group, and 558 patients were grouped into the DSW–Married group. Married–Married group patients were more likely to have private insurance (80% vs 72% and 68%), to be white (83% vs 79% and 68%) and to have a lower stage tumour than DSW–DSW group patients (Appendix Table 3) and Single–Single group patients (Appendix Table 1). Married–Married group patients were older than Single–Single group patients (median age 62 years vs 57 years; Appendix Table 1) but younger than DSW–DSW group patients (median age 62 years vs 72 years; Appendix Table 3). Compared to Single–Married group patients, Single–Single group patients were older (median age 57 years vs 53 years), less frequently white (74% and 68%), and less likely to have private insurance (68% vs 74%) (Appendix Table 4). Married–DSW group patients were older (median age 71 years vs 62 years), less likely to have private insurance (77% vs 80%), and had a lower stage tumour (68% vs 73%) compared to Married–Married group patients (Appendix Table 5). In contrast to DSW–Married group patients, DSW–DSW group patients were older (median age 72 years vs 63 years), less likely to have private insurance (72% vs 78%), and had a lower stage tumour (71% vs 74%) (Appendix Table 6).

**Table 1:** Baseline demographic and tumor characteristics of patients according to marital status in SEER database.

**Characteristics**	Total	Single-single	Single-married	Single-DSW	Married-married	Married-DSW	DSW–DSW	DSW-married
	N = 33,089	N = 3306	N = 540	N = 686	N = 17,623	N = 3043	N = 7333	N = 558
	No (%)	No (%)	No (%)	No (%)	No (%)	No (%)	No (%)	No (%)
**Year of diagnosis**								
1992–1997	1191 (4)	116 (4)	8 (1)	9 (1)	651 (4)	46 (2)	347 (5)	14 (3)
1998–2003	4761 (14)	483 (15)	67 (12)	88 (13)	2538 (14)	341 (11)	1167 (16)	77 (14)
2004–2009	10,686 (32)	1062 (32)	190 (35)	218 (32)	5707 (32)	927 (30)	2400 (33)	182 (33)
2010–2015	16,451 (50)	1645 (50)	275 (51)	371 (54)	8727 (50)	1729 (57)	3419 (47)	285 (51)
**Race**								
White	26,502 (80)	2235 (68)	396 (74)	496 (72)	14,661 (83)	2483 (82)	5792 (79)	439 (79)
Black	3777 (11)	786 (24)	82 (15)	160 (23)	1249 (7)	326 (11)	1086 (15)	88 (16)
Other	2800 (9)	285 (9)	60 (11)	30 (4)	1711 (10)	234 (8)	449 (6)	31 (6)
Unknown	10 (0)	0 (0)	2 (0)	0 (0)	2 (0)	0 (0)	6 (0)	0 (0)
**Insurance**								
Private insurance	17,120 (52)	1460 (44)	290 (54)	318 (46)	9674 (55)	1761 (58)	3308 (45)	309 (55)
Insured/no specifics	3151 (10)	310 (9)	53 (10)	72 (10)	1579 (9)	312 (10)	764 (10)	61 (11)
Any medicaid	1985 (6)	436 (13)	43 (8)	91 (13)	544 (3)	207 (7)	642 (9)	22 (4)
Uninsured	185 (1)	42 (1)	6 (1)	6 (1)	81 (0)	20 (1)	25 (0)	5 (1)
Unknown	10,648 (32)	1058 (32)	148 (27)	199 (29)	5745 (33)	743 (24)	2594 (35)	161 (29)
**Grade**								
I	7133 (22)	645 (20)	89 (16)	142 (21)	3824 (22)	653 (21)	1653 (23)	127 (23)
II	13,418 (41)	1288 (39)	203 (38)	282 (41)	7096 (40)	1282 (42)	3055 (42)	212 (38)
III	10,028 (30)	1116 (34)	201 (37)	213 (31)	5369 (30)	912 (30)	2036 (28)	181 (32)
Unknown	2510 (8)	257 (8)	47 (9)	49 (7)	1334 (8)	196 (6)	589 (8)	38 (7)
**Histology**								
IDC	22,999 (70)	2329 (70)	386 (71)	458 (67)	12,283 (70)	2158 (71)	4982 (68)	403 (72)
ILC	3528 (11)	328 (10)	39 (7)	76 (11)	1910 (11)	314 (10)	806 (11)	55 (10)
Other	6562 (20)	649 (20)	115 (21)	152 (22)	3430 (19)	571 (19)	1545 (21)	100 (18)
**AJCC T stage**								
pT1	23,672 (72)	2248 (68)	346 (64)	459 (67)	12,923 (73)	2077 (68)	5208 (71)	411 (74)
pT2	6347 (19)	687 (21)	125 (23)	151 (22)	3225 (18)	633 (21)	1425 (19)	101 (18)
pT3	1057 (3)	125 (4)	27 (5)	17 (2)	509 (3)	108 (4)	253 (3)	18 (3)
pT4	861 (3)	104 (3)	17 (3)	26 (4)	403 (2)	92 (3)	208 (3)	11 (2)
Any T, Mets	1152 (3)	142 (4)	25 (5)	33 (5)	563 (3)	133 (4)	239 (3)	17 (3)
**AJCC N stage**								
pN0	25,844 (78)	2552 (77)	411 (76)	530 (77)	13,757 (78)	2381 (78)	5775 (79)	438 (78)
pN1	4832 (15)	473 (14)	87 (16)	95 (14)	2621 (15)	434 (14)	1046 (14)	76 (14)
pN2	1217 (4)	145 (4)	21 (4)	23 (3)	611 (3)	124 (4)	268 (4)	25 (4)
pN3	1196 (4)	136 (4)	21 (4)	38 (6)	634 (4)	104 (3)	244 (3)	19 (3)
**ER**								
Negative	6611 (20)	714 (22)	148 (27)	143 (21)	3549 (20)	610 (20)	1344 (18)	103 (18)
Positive	24,129 (73)	2319 (70)	365 (68)	495 (72)	12,870 (73)	2272 (75)	5390 (74)	418 (75)
Unknown	2349 (7)	273 (8)	27 (5)	48 (7)	1204 (7)	161 (5)	599 (8)	37 (7)
**PR**								
Negative	10,986 (33)	1167 (35)	201 (37)	246 (36)	5906 (34)	1012 (33)	2283 (31)	171 (31)
Positive	19,329 (58)	1840 (56)	310 (57)	382 (56)	10,257 (58)	1845 (61)	4353 (59)	342 (61)
Unknown	2774 (8)	299 (9)	29 (5)	58 (8)	1460 (8)	186 (6)	697 (10)	45 (8)
**Surgery**								
Nonsurgery	1540 (5)	204 (6)	31 (6)	56 (8)	664 (4)	179 (6)	378 (5)	28 (5)
BCS	12,412 (38)	1187 (36)	166 (31)	237 (35)	6419 (36)	1168 (38)	3011 (41)	224 (40)
Mastectomy	19,120 (58)	1913 (58)	343 (64)	392 (57)	10,534 (60)	1694 (56)	3939 (54)	305 (55)
Unknown	17 (0)	2 (0)	0 (0)	1 (0)	6 (0)	2 (0)	5 (0)	1 (0)
**Radiotherapy**								
No	22,381 (68)	2260 (68)	377 (70)	461 (67)	11,761 (67)	2063 (68)	5091 (69)	368 (66)
Yes	10,073 (30)	980 (30)	149 (28)	210 (31)	5517 (31)	920 (30)	2124 (29)	173 (31)
Unknown	635 (2)	66 (2)	14 (3)	15 (2)	345 (2)	60 (2)	118 (2)	17 (3)
**Chemotherapy**								
No	23,373 (71)	2183 (66)	317 (59)	471 (69)	11,973 (68)	2280 (75)	5770 (79)	379 (68)
Yes	9716 (29)	1123 (34)	223 (41)	215 (31)	5650 (32)	763 (25)	1563 (21)	179 (32)
**Age (years)**								
20–40	743 (2)	190 (6)	53 (10)	14 (2)	385 (2)	43 (1)	42 (1)	16 (3)
40–50	3991 (12)	654 (20)	157 (29)	62 (9)	2469 (14)	221 (7)	366 (5)	62 (11)
50–65	11,961 (36)	1424 (43)	228 (42)	268 (39)	7326 (42)	768 (25)	1700 (23)	247 (44)
≥ 65	16,394 (50)	1038 (31)	102 (19)	342 (50)	7443 (42)	2011 (66)	5225 (71)	233 (42)
Median (range)	64 (55,74)	57 (49,67)	53 (45,61)	64 (55.25,73)	62 (53,71)	71 (60,79)	72 (63,80)	63 (54,70.75)
**Interval from primary to SBC, median (interquartile range), months**								
	73 (33,120)	62 (26,109)	88 (51,128.25)	81 (44,129)	72 (32, 119)	106 (66,156)	62 (26,106)	90.5 (50.25,135)

### Effects of marital status on BCSS and OS

**Figure 2:**
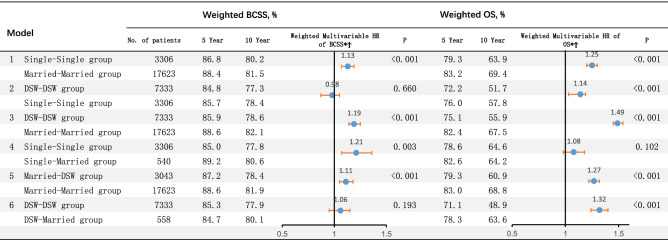
Hazard ratio comparing BCSS and OS according to marital status. (*) Weighted by inverse propensity score. (Ϯ) Multivariate analysis adjusted by year of diagnosis, age, race, year of diagnosis, insurance, histology type, nuclear grade, T stage, N stage, M stage, ER status, PR status, surgery, chemotherapy and radiotherapy. *BCSS* breast cancer-caused special survival, *OS* overall survival, *HR* hazard ratio, *DSW* divorced/separated/widowed.

A total of 4619 deaths due to breast cancer (14.0%) and 4796 deaths resulting from other cancers (14.5%) were identified in the cohort. The median recurrence time was 64 months (interquartile range, 55 to 74 months). Six models (Model 1, Single–Single group vs Married–Married group; Model 2, DSW–DSW group vs Single–Single group; Model 3, DSW–DSW group vs Married–Married group; Model 4, Single–Single group vs Single–Married group; Model 5, Married–DSW group vs Married–Married group; Model 6, DSW–DSW group vs DSW–Married group) were constructed to examine the association between dynamic changes in marital status and survival benefit. Considering the possible interaction among variables, we conducted multivariate Cox regression analysis adjusting for year of diagnosis, age, race, insurance, histology type, nuclear grade, T stage, N stage, M stage, ER status, PR status, surgery, chemotherapy and radiotherapy. After adjusting for clinical factors and considering the propensity score, it was found that compared with the Single–Single group and DSW–DSW group patients, the patients in the Married–Married group were significantly associated with better BCSS (HR 1.13, 95% CI: 1.03–1.19, *P* < 0.001; HR 1.19, 95% CI: 1.14–1.25, *P* < 0.001, respectively) and OS (HR 1.25, 95% CI: 1.20–1.30, *P* < 0.001; HR 1.49, 95% CI: 1.45–1.54, *P* < 0.001, respectively). In contrast to the DSW–DSW group, the patients in the Single–Single group and the DSW–Married group showed similar BCSS (HR 0.98, 95% CI: 0.92–1.05, *P* = 0.660; HR 1.06, 95% CI: 0.97–1.15, *P* = 0.193, respectively) but better OS (HR 1.14, 95% CI: 1.09–1.19, *P* < 0.001; HR 1.32, 95% CI: 1.25–1.40, *P* < 0.001, respectively). Compared with the Single–Single group, the Single–Married group exhibited significantly better BCSS (HR 1.21, 95% CI: 1.07–1.36, *P* = 0.003) but showed no difference in OS (HR 1.08, 95% CI: 0.98–1.18, *P* = 0.102). In contrast to the Married–DSW group, the Married–Married group showed better BCSS (HR 1.11, 95% CI: 1.05–1.18, *P* < 0.001) and OS (HR 1.27, 95% CI: 1.22–1.32, *P* < 0.001) (Fig. 2).

**Figure 3:**
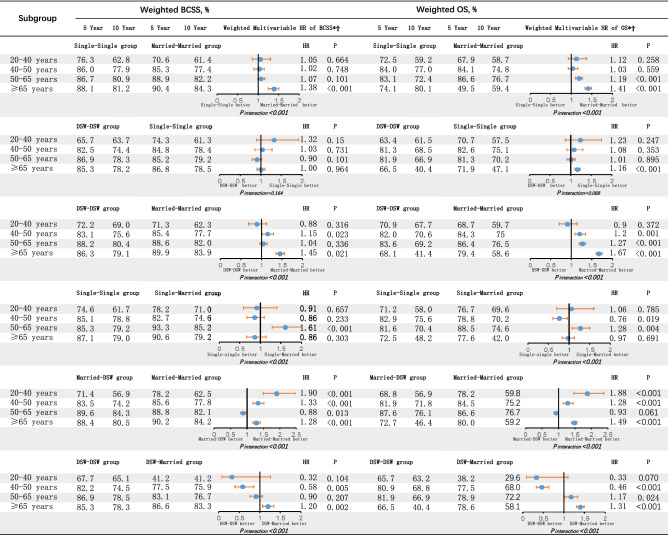
Hazard ratio comparing BCSS and OS according to marital status in different subgroups. (*) Weighted by inverse propensity score. (Ϯ) Multivariate analysis adjusted by year of diagnosis, age, race, year of diagnosis, insurance, histology type, nuclear grade, T stage, N stage, M stage, ER status, PR status, surgery, chemotherapy and radiotherapy. *BCSS* breast cancer-caused special survival, *OS* overall survival, *HR* hazard ratio, *DSW* divorced/separated/widowed.

In addition, the effect of marital status varied depending upon age. In general, the older the BC patients were, the greater the survival benefit they had. For younger patients aged 20 to 40 years, except for model 5, the other model results suggested that marital status was not significantly associated with BCSS or OS (Fig. 2). However, for older patients (≥ 65 years), the majority of model results proved that marital status was significantly associated with BCSS and OS. In addition, for patients in the ranges of 40–50 years and 50–65 years, the findings were mixed. Some results showed that these patients received a survival benefit from marriage, while other results did not. Furthermore, some results showed that even present marriage would produce negative effects on BCSS and OS (Fig. 3).

Sensitivity analyses were performed after excluding variables including insurance, ER, and PR status. Repeating the analyses using the proportional subdistribution hazards model and using random survival forest methodology after multiple imputation of unknown data, we observed similar findings.

## Discussion

This SEER analysis is a pioneering study to specifically examine whether dynamic changes in marital status have a significant impact on the survival rate of BC patients. In our study, we observed a remarkably higher risk of death among unmarried SBC patients than among married SBC patients. A noticeable benefit to OS from positive marital status was observed in five models, while that to BCSS occurred in only four models. Previous studies have revealed that a single person has an increased risk of breast cancer, and single patients without a marriage or childbearing history might have worse survival outcomes than DSW patients^19^. In our study, after controlling the possible interactions between variables with multivariate analysis, we discovered that the patients in the Single–Single group displayed no worse BCSS and OS than did those in the DSW–DSW group. This result suggests that the survival benefit of marriage is more due to social and emotional support than to full-term pregnancy.

A growing body of evidence shows that marital status acts as an independent prognostic factor for survival in patients with breast cancer. The influence of marital status on prognosis might be related to tumour stage, receipt of treatment, and social support^3,20–22^. In our study, we found that married patients in each model had a significantly higher prevalence of white race, private insurance and early tumour stage, which would improve their prognosis. Women's social relationship influenced by marital status has been proven to impact the choice of mastectomy or breast-conserving surgery whether to receive chemotherapy, and other treatment decisions^23–26^. Our study produced similar results in that the likelihood of receiving radiotherapy, chemotherapy, and surgeries among unmarried groups in each model was lower than that among married groups. Moreover, other studies also showed that a supportive spouse could have a positive effect on his partner’s health-seeking behaviour. For instance, investigators have shown that married individuals tend to perform basic and simple healthy behaviours, such as eating more balanced meals and exercising more than unmarried individuals^27,28^. These behaviours can explain why women with a positive marital relationship are less likely to contract breast cancer. In our study, when the patient's marital status changed from single or DSW to married, the median interval time from primary BC to SBC became longer. Although Married–DSW group patients had a longer median interval time than Married–Married group patients, the tumour stage of Married–DSW group patients was more advanced, which suggested that the Married–DSW group patients were not screened for breast cancer in time (Table 1). Cancer screening is a generally effective strategy to detect tumours in the early stage, improve quality of life and reduce costs in health care^29–31^. Hanske et al. claimed that more unfavourable outcomes in unmarried individuals might be linked to a lack of cancer screening in this group^32^. Considering the increasing rate of more advanced stages amongst unmarried patients, it may be hypothesized that cancer screening is less frequent among such patients, which could affect their BCSS and OS.

Furthermore, we discovered an interesting phenomenon in which the survival benefit of marriage was evident in older patients but not in younger patients. This may be because BC treatments are usually more aggressive in young patients, which could result in higher levels of psychological distress, such as chemotherapy-induced menopause and fertility issues. This can impact the couple relationship and, consequently, patient survival^33,34^. Croft et al. highly emphasized that marital status significantly impacted the short-term survival benefit more than the long-term survival benefit. This may be because compared with short-term survivors, long-term breast cancer survivors are more likely to be able to resume usual activities and have more time to adjust to their situation. On the other hand, short-term survivors may spend more time at home and seek more assistance from their spouse, partner, or other individuals for daily living activities^35^. These results demonstrate that oncologists and other cancer providers should recognize that unmarried patients (especially short-term survivors), a high-risk group for higher mortality, need more social and psychological support.

Subject to restrictions on our knowledge and research methods, this study has several limitations. The first limitation is that the biases presented in any retrospective study may increase the bias of the results. Second, although assessing marital status at the time of diagnosis of primary BC and SBC is appropriate, we still cannot assess the change in marital status after the breast cancer diagnosis. In addition, it is also important to note that we lack information on the quality of marriage and of support from children and other family members. We also lack information on specific treatments and comorbidities. This information could be a potential confounder in our analyses. Finally, we cannot dismiss the possibility of self-selection in that women who are physically, emotionally, or psychologically healthier may be more likely to marry than their counterparts, which will affect the reliability of the results.

## Conclusion

Our study demonstrated that, regardless of their previous marital status, married patients had a better prognosis than unmarried patients. Meanwhile, single patients obtained better survival outcomes than divorced/separated/widowed patients. These effects were more significant in older people. For physicians, our results highlight the necessity to proactively provide single and divorced/separated/widowed individuals with appropriate social and psychological support that would benefit them.

## Supplementary Information

Supplementary Table 1.

Supplementary Table 2.

Supplementary Table 3.

Supplementary Table 4.

Supplementary Table 5.

Supplementary Table 6.
