# Supplementary material for: Dynamic changes in marital status and survival in women with breast cancer: a population-based study
Source: Sci Rep. 2021 Mar 8;11:5421. doi: 10.1038/s41598-021-84996-y (PMC7940486; doi:10.1038/s41598-021-84996-y)
Supplement: Supplementary file 1 — Supplementary Table 1. [file 41598_2021_84996_MOESM1_ESM.docx]

Appendix table 1. Baseline demographic and tumor characteristics of patients according to marital status in SEER database.

|  | **Unmatched** | |  | **Matched** | |
| --- | --- | --- | --- | --- | --- |
|  | **No. of patients (%)** | |  | **No. of patients (%)** | |
| **Characteristic** | **Married-Married**  **N=17623** | **Single-Single**  **N=3306** | **P** | **Married-Married**  **N=17623** | **Single-Single**  **N=3306** |
| **Year of diagnosis** |  |  |  |  |  |
| 1992-1997 | 651(4) | 116(4) | 0.933 | 654(4) | 125(4) |
| 1998-2003 | 2538(14) | 483(15) | 0.933 | 2539(14) | 508(15) |
| 2004-2009 | 5707(32) | 1062(32) | 0.933 | 5715(32) | 1073(32) |
| 2010-2015 | 8727(50) | 1645(50) | 0.933 | 8715(49) | 1600(48) |
| **Race** |  |  |  |  |  |
| White | 14662(83) | 2235(68) | <.001 | 14419(82) | 2498(76) |
| Black | 1249(7) | 786(24) | <.001 | 1313(7) | 613(19) |
| Other | 1712(10) | 285(9) | <.001 | 1890(11) | 195(6) |
| **Insurance** |  |  |  |  |  |
| Private insurance | 14062(80) | 2253(68) | <.001 | 13752(78) | 2581(78) |
| Insured/no specifics | 2383(14) | 457(14) | <.001 | 2482(14) | 364(11) |
| Any Medicaid | 1048(6) | 541(16) | <.001 | 1266(7) | 287(9) |
| Uninsured | 130(1) | 55(2) | <.001 | 123(1) | 75(2) |
| **Grade** |  |  |  |  |  |
| I | 4139(23) | 707(21) | <.001 | 4076(23) | 776(23) |
| II | 7681(44) | 1397(42) | <.001 | 7656(43) | 1424(43) |
| III | 5803(33) | 1202(36) | <.001 | 5891(33) | 1106(33) |
| **Histology** |  |  |  |  |  |
| IDC | 12283(70) | 2329(70) | 0.293 | 12296(70) | 2298(70) |
| ILC | 1910(11) | 328(10) | 0.293 | 1892(11) | 351(11) |
| Other | 3430(19) | 649(20) | 0.293 | 3436(19) | 656(19) |
| **AJCC T Stage** |  |  |  |  |  |
| pT1 | 12923(73) | 2248(68) | <.001 | 12786(73) | 2364(72) |
| pT2 | 3225(18) | 687(21) | <.001 | 3273(19) | 649(20) |
| pT3 | 509(3) | 125(4) | <.001 | 530(3) | 102(3) |
| pT4 | 403(2) | 104(3) | <.001 | 439(2) | 75(2) |
| Any T, Mets | 563(3) | 142(4) | <.001 | 595(3) | 117(4) |
| **AJCC N Stage** |  |  |  |  |  |
| pN0 | 13757(78) | 2552(77) | 0.024 | 13717(78) | 2605(79) |
| pN1 | 2621(15) | 473(14) | 0.024 | 2639(15) | 439(13) |
| pN2 | 611(3) | 145(4) | 0.024 | 615(3) | 138(4) |
| pN3 | 634(4) | 136(4) | 0.024 | 652(4) | 124(4) |
| **ER** |  |  |  |  |  |
| Negative | 3860(22) | 782(24) | 0.028 | 3908(22) | 725(22) |
| Positive | 13763(78) | 2524(76) | 0.028 | 13715(78) | 2581(78) |
| **PR** |  |  |  |  |  |
| Negative | 6452(37) | 1279(39) | 0.024 | 6509(37) | 1212(37) |
| Positive | 11171(63) | 2027(61) | 0.024 | 11114(63) | 2094(63) |
| **Surgery** |  |  |  |  |  |
| Nonsurgery | 666(4) | 204(6) | <.001 | 730(4) | 140(4) |
| BCS | 6419(36) | 1188(36) | <.001 | 6409(36) | 1182(36) |
| Mastectomy | 10538(60) | 1914(58) | <.001 | 10484(60) | 1985(60) |
| **Radiotherapy** |  |  |  |  |  |
| No | 11955(68) | 2301(70) | 0.048 | 12006(68) | 2259(68) |
| Yes | 5668(32) | 1005(30) | 0.048 | 5617(32) | 1047(32) |
| **Chemotherapy** |  |  |  |  |  |
| No | 11973(68) | 2183(66) | 0.033 | 11926(68) | 2254(68) |
| Yes | 5650(32) | 1123(34) | 0.033 | 5697(32) | 1052(32) |
| **Age (years)** |  |  |  |  |  |
| 20-40 | 385(2) | 190(6) | <.001 | 458(3) | 104(3) |
| 40-50 | 2469(14) | 654(20) | <.001 | 2673(15) | 477(14) |
| 50-65 | 7326(42) | 1424(43) | <.001 | 7362(42) | 1378(42) |
| ≥65 | 7443(42) | 1038(31) | <.001 | 7131(40) | 1347(41) |
