# Supplementary material for: Dynamic changes in marital status and survival in women with breast cancer: a population-based study
Source: Sci Rep. 2021 Mar 8;11:5421. doi: 10.1038/s41598-021-84996-y (PMC7940486; doi:10.1038/s41598-021-84996-y)
Supplement: Supplementary file 2 — Supplementary Table 2. [file 41598_2021_84996_MOESM2_ESM.docx]

Appendix table 2. Baseline demographic and tumor characteristics of patients according to marital status in SEER database.

|  | **Unmatched** | |  | **Matched** | |
| --- | --- | --- | --- | --- | --- |
|  | **No. of patients (%)** | |  | **No. of patients (%)** | |
| **Characteristic** | **DSW-DSW**  **N=7333** | **Single-Single**  **N=3306** | **P** | **DSW-DSW**  **N=7333** | **Single-Single**  **N=3306** |
| **Year of diagnosis** |  |  |  |  |  |
| 1992-1997 | 347(5) | 116(4) | 0.002 | 334(5) | 145(4) |
| 1998-2003 | 1167(16) | 483(15) | 0.002 | 1153(16) | 526(16) |
| 2004-2009 | 2400(33) | 1062(32) | 0.002 | 2380(32) | 1064(32) |
| 2010-2015 | 3419(47) | 1645(50) | 0.002 | 3466(47) | 1571(48) |
| **Race** |  |  |  |  |  |
| White | 5798(79) | 2235(68) | <.001 | 5556(76) | 2470(75) |
| Black | 1086(15) | 786(24) | <.001 | 1212(17) | 610(18) |
| Other | 449(6) | 285(9) | <.001 | 564(7) | 226(7) |
| **Insurance** |  |  |  |  |  |
| Private insurance | 5283(72) | 2253(68) | <.001 | 5191(71) | 2303(70) |
| Insured/no specifics | 1143(16) | 457(14) | <.001 | 1100(15) | 487(15) |
| Any Medicaid | 867(12) | 541(16) | <.001 | 993(14) | 478(14) |
| Uninsured | 40(1) | 55(2) | <.001 | 49(1) | 38(1) |
| **Grade** |  |  |  |  |  |
| I | 1807(25) | 707(21) | <.001 | 1720(23) | 775(23) |
| II | 3320(45) | 1397(42) | <.001 | 3229(44) | 1474(45) |
| III | 2206(30) | 1202(36) | <.001 | 2383(33) | 1057(32) |
| **Histology** |  |  |  |  |  |
| IDC | 4982(68) | 2329(70) | 0.033 | 5043(69) | 2285(69) |
| ILC | 806(11) | 328(10) | 0.033 | 802(11) | 372(11) |
| Other | 1545(21) | 649(20) | 0.033 | 1488(20) | 649(20) |
| **AJCC T Stage** |  |  |  |  |  |
| pT1 | 5208(71) | 2248(68) | 0.01 | 5135(70) | 2309(70) |
| pT2 | 1425(19) | 687(21) | 0.01 | 1435(20) | 668(20) |
| pT3 | 253(3) | 125(4) | 0.01 | 265(4) | 111(3) |
| pT4 | 208(3) | 104(3) | 0.01 | 219(3) | 95(3) |
| Any T, Mets | 239(3) | 142(4) | 0.01 | 279(4) | 124(4) |
| **AJCC N Stage** |  |  |  |  |  |
| pN0 | 5775(79) | 2552(77) | 0.05 | 5712(78) | 2604(79) |
| pN1 | 1046(14) | 473(14) | 0.05 | 1066(15) | 443(13) |
| pN2 | 268(4) | 145(4) | 0.05 | 290(4) | 133(4) |
| pN3 | 244(3) | 136(4) | 0.05 | 264(4) | 127(4) |
| **ER** |  |  |  |  |  |
| Negative | 1484(20) | 782(24) | <.001 | 1589(22) | 700(21) |
| Positive | 5849(80) | 2524(76) | <.001 | 5744(78) | 2606(79) |
| **PR** |  |  |  |  |  |
| Negative | 2528(34) | 1279(39) | <.001 | 2620(36) | 1176(36) |
| Positive | 4805(66) | 2027(61) | <.001 | 4713(64) | 2130(64) |
| **Surgery** |  |  |  |  |  |
| Nonsurgery | 378(5) | 204(6) | <.001 | 392(5) | 190(6) |
| BCS | 3014(41) | 1188(36) | <.001 | 2884(39) | 1256(38) |
| Mastectomy | 3941(54) | 1914(58) | <.001 | 4057(55) | 1860(56) |
| **Radiotherapy** |  |  |  |  |  |
| No | 5153(70) | 2301(70) | 0.499 | 5129(70) | 2301(70) |
| Yes | 2180(30) | 1005(30) | 0.499 | 2204(30) | 1005(30) |
| **Chemotherapy** |  |  |  |  |  |
| No | 5770(79) | 2183(66) | <.001 | 5435(74) | 2439(74) |
| Yes | 1563(21) | 1123(34) | <.001 | 1898(26) | 867(26) |
| **Age (years)** |  |  |  |  |  |
| 20-40 | 42(1) | 190(6) | <.001 | 214(3) | 69(2) |
| 40-50 | 366(5) | 654(20) | <.001 | 794(11) | 302(9) |
| 50-65 | 1700(23) | 1424(43) | <.001 | 1993(27) | 1086(33) |
| ≥65 | 5225(71) | 1038(31) | <.001 | 4332(59) | 1848(56) |
