# Supplementary material for: Dynamic changes in marital status and survival in women with breast cancer: a population-based study
Source: Sci Rep. 2021 Mar 8;11:5421. doi: 10.1038/s41598-021-84996-y (PMC7940486; doi:10.1038/s41598-021-84996-y)
Supplement: Supplementary file 3 — Supplementary Table 3. [file 41598_2021_84996_MOESM3_ESM.docx]

Appendix table 3. Baseline demographic and tumor characteristics of patients according to marital status in SEER database.

|  | **Unmatched** | |  | **Matched** | |
| --- | --- | --- | --- | --- | --- |
|  | **No. of patients (%)** | |  | **No. of patients (%)** | |
| **Characteristic** | **DSW-DSW**  **N=7333** | **Married-Married**  **N=17623** | **P** | **DSW-DSW**  **N=7333** | **Married-Married**  **N=17623** |
| **Year of diagnosis** |  |  |  |  |  |
| 1992-1997 | 347(5) | 651(4) | <.001 | 313(4) | 701(4) |
| 1998-2003 | 1167(16) | 2538(14) | <.001 | 1142(16) | 2664(15) |
| 2004-2009 | 2400(33) | 5707(32) | <.001 | 2400(33) | 5698(32) |
| 2010-2015 | "3419(47)" | 8727(50) | <.001 | 3478(47) | 8560(49) |
| **Race** |  |  |  |  |  |
| White | 5798(79) | 14662(83) | <.001 | 5779(79) | 14644(83) |
| Black | 1086(15) | 1249(7) | <.001 | 1113(15) | 1244(7) |
| Other | 449(6) | 1712(10) | <.001 | 441(6) | 1734(10) |
| **Insurance** |  |  |  |  |  |
| Private insurance | 5283(72) | 14062(80) | <.001 | 5680(77) | 13556(77) |
| Insured/no specifics | 1143(16) | 2383(14) | <.001 | 889(12) | 2677(15) |
| Any Medicaid | 867(12) | 1048(6) | <.001 | 683(9) | 1280(7) |
| Uninsured | 40(1) | 130(1) | <.001 | 81(1) | 110(1) |
| **Grade** |  |  |  |  |  |
| I | 1807(25) | 4139(23) | <.001 | 1715(23) | 4204(24) |
| II | 3320(45) | 7681(44) | <.001 | 3218(44) | 7747(44) |
| III | 2206(30) | 5803(33) | <.001 | 2400(33) | 5673(32) |
| **Histology** |  |  |  |  |  |
| IDC | 4982(68) | 12283(70) | 0.01 | 5121(70) | 12200(69) |
| ILC | 806(11) | 1910(11) | 0.01 | 785(11) | 1940(11) |
| Other | 1545(21) | 3430(19) | 0.01 | 1427(19) | 3482(20) |
| **AJCC T Stage** |  |  |  |  |  |
| pT1 | 5208(71) | 12923(73) | 0.001 | 5322(73) | 12827(73) |
| pT2 | 1425(19) | 3225(18) | 0.001 | 1370(19) | 3252(18) |
| pT3 | 253(3) | 509(3) | 0.001 | 234(3) | 528(3) |
| pT4 | 208(3) | 403(2) | 0.001 | 161(2) | 445(3) |
| Any T, Mets | 239(3) | 563(3) | 0.001 | 246(3) | 570(3) |
| **AJCC N Stage** |  |  |  |  |  |
| pN0 | 5775(79) | 13757(78) | 0.36 | 5699(78) | 13807(78) |
| pN1 | 1046(14) | 2621(15) | 0.36 | 1088(15) | 2572(15) |
| pN2 | 268(4) | 611(3) | 0.36 | 305(4) | 600(3) |
| pN3 | 244(3) | 634(4) | 0.36 | 242(3) | 644(4) |
| **ER** |  |  |  |  |  |
| Negative | 1484(20) | 3860(22) | 0.004 | 1623(22) | 3780(21) |
| Positive | 5849(80) | 13763(78) | 0.004 | 5710(78) | 13843(79) |
| **PR** |  |  |  |  |  |
| Negative | 2528(34) | 6452(37) | 0.001 | 2657(36) | 6348(36) |
| Positive | 4805(66) | 11171(63) | 0.001 | 4676(64) | 11275(64) |
| **Surgery** |  |  |  |  |  |
| Nonsurgery | 378(5) | 666(4) | <.001 | 294(4) | 750(4) |
| BCS | 3014(41) | 6419(36) | <.001 | 2794(38) | 6646(38) |
| Mastectomy | 3941(54) | 10538(60) | <.001 | 4245(58) | 10227(58) |
| **Radiotherapy** |  |  |  |  |  |
| No | 5153(70) | 11955(68) | <.001 | 4963(68) | 12050(68) |
| Yes | 2180(30) | 5668(32) | <.001 | 2370(32) | 5573(32) |
| **Chemotherapy** |  |  |  |  |  |
| No | 5770(79) | 11973(68) | <.001 | 5080(69) | 12504(71) |
| Yes | 1563(21) | 5650(32) | <.001 | 2253(31) | 5119(29) |
| **Age (years)** |  |  |  |  |  |
| 20-40 | 42(1) | 385(2) | <.001 | 239(3) | 286(2) |
| 40-50 | 366(5) | 2469(14) | <.001 | 1032(14) | 1949(11) |
| 50-65 | 1700(23) | 7326(42) | <.001 | 2326(32) | 6560(37) |
| ≥65 | 5225(71) | 7443(42) | <.001 | 3737(51) | 8828(50) |
