# Supplementary material for: Dynamic changes in marital status and survival in women with breast cancer: a population-based study
Source: Sci Rep. 2021 Mar 8;11:5421. doi: 10.1038/s41598-021-84996-y (PMC7940486; doi:10.1038/s41598-021-84996-y)
Supplement: Supplementary file 4 — Supplementary Table 4. [file 41598_2021_84996_MOESM4_ESM.docx]

Appendix table 4. Baseline demographic and tumor characteristics of patients according to marital status in SEER database.

|  | **Unmatched** | |  | **Matched** | |
| --- | --- | --- | --- | --- | --- |
|  | **No. of patients (%)** | |  | **No. of patients (%)** | |
| **Characteristic** | **Single-Married**  **N=540** | **Single-Single**  **N=3306** | **P** | **Single-Married**  **N=540** | **Single-Single**  **N=3306** |
| **Year of diagnosis** |  |  |  |  |  |
| 1992-1997 | 8(1) | 116(4) | 0.03 | 12(2) | 112(3) |
| 1998-2003 | 67(12) | 483(15) | 0.03 | 79(15) | 475(14) |
| 2004-2009 | 190(35) | 1062(32) | 0.03 | 192(36) | 1057(32) |
| 2010-2015 | 275(51) | 1645(50) | 0.03 | 258(48) | 1663(50) |
| **Race** |  |  |  |  |  |
| White | 397(74) | 2235(68) | <.001 | 392(73) | 2242(68) |
| Black | 83(15) | 786(24) | <.001 | 81(15) | 788(24) |
| Other | 60(11) | 285(9) | <.001 | 67(12) | 276(8) |
| **Insurance** |  |  |  |  |  |
| Private insurance | 401(74) | 2253(68) | 0.007 | 375(69) | 2278(69) |
| Insured/no specifics | 72(13) | 457(14) | 0.007 | 86(16) | 446(13) |
| Any Medicaid | 58(11) | 541(16) | 0.007 | 74(14) | 521(16) |
| Uninsured | 9(2) | 55(2) | 0.007 | 5(1) | 61(2) |
| **Grade** |  |  |  |  |  |
| I | 100(19) | 707(21) | 0.152 | 108(20) | 697(21) |
| II | 223(41) | 1397(42) | 0.152 | 240(44) | 1386(42) |
| III | 217(40) | 1202(36) | 0.152 | 192(36) | 1223(37) |
| **Histology** |  |  |  |  |  |
| IDC | 386(71) | 2329(70) | 0.119 | 387(72) | 2329(70) |
| ILC | 39(7) | 328(10) | 0.119 | 43(8) | 325(10) |
| Other | 115(21) | 649(20) | 0.119 | 110(20) | 652(20) |
| **AJCC T Stage** |  |  |  |  |  |
| pT1 | 346(64) | 2248(68) | 0.385 | 368(68) | 2234(68) |
| pT2 | 125(23) | 687(21) | 0.385 | 112(21) | 693(21) |
| pT3 | 27(5) | 125(4) | 0.385 | 23(4) | 129(4) |
| pT4 | 17(3) | 104(3) | 0.385 | 14(3) | 107(3) |
| Any T, Mets | 25(5) | 142(4) | 0.385 | 22(4) | 143(4) |
| **AJCC N Stage** |  |  |  |  |  |
| pN0 | 411(76) | 2552(77) | 0.698 | 412(76) | 2552(77) |
| pN1 | 87(16) | 473(14) | 0.698 | 87(16) | 472(14) |
| pN2 | 21(4) | 145(4) | 0.698 | 19(3) | 146(4) |
| pN3 | 21(4) | 136(4) | 0.698 | 22(4) | 136(4) |
| **ER** |  |  |  |  |  |
| Negative | 152(28) | 782(24) | 0.028 | 133(25) | 803(24) |
| Positive | 388(72) | 2524(76) | 0.028 | 407(75) | 2503(76) |
| **PR** |  |  |  |  |  |
| Negative | 208(39) | 1279(39) | 0.978 | 211(39) | 1279(39) |
| Positive | 332(61) | 2027(61) | 0.978 | 329(61) | 2027(61) |
| **Surgery** |  |  |  |  |  |
| Nonsurgery | 31(6) | 204(6) | 0.045 | 36(6) | 200(6) |
| BCS | 166(31) | 1188(36) | 0.045 | 187(35) | 1168(35) |
| Mastectomy | 343(64) | 1914(58) | 0.045 | 316(59) | 1938(59) |
| **Radiotherapy** |  |  |  |  |  |
| No | 384(71) | 2301(70) | 0.51 | 375(69) | 2308(70) |
| Yes | 156(29) | 1005(30) | 0.51 | 165(31) | 998(30) |
| **Chemotherapy** |  |  |  |  |  |
| No | 317(59) | 2183(66) | 0.001 | 352(65) | 2148(65) |
| Yes | 223(41) | 1123(34) | 0.001 | 188(35) | 1158(35) |
| **Age (years)** |  |  |  |  |  |
| 20-40 | 53(10) | 190(6) | <.001 | 30(6) | 216(7) |
| 40-50 | 157(29) | 654(20) | <.001 | 122(23) | 690(21) |
| 50-65 | 228(42) | 1424(43) | <.001 | 226(42) | 1415(43) |
| ≥65 | 102(19) | 1038(31) | <.001 | 162(30) | 985(30) |
