# Supplementary material for: Dynamic changes in marital status and survival in women with breast cancer: a population-based study
Source: Sci Rep. 2021 Mar 8;11:5421. doi: 10.1038/s41598-021-84996-y (PMC7940486; doi:10.1038/s41598-021-84996-y)
Supplement: Supplementary file 5 — Supplementary Table 5. [file 41598_2021_84996_MOESM5_ESM.docx]

Appendix table 5. Baseline demographic and tumor characteristics of patients according to marital status in SEER database.

|  | **Unmatched** | |  | **Matched** | |
| --- | --- | --- | --- | --- | --- |
|  | **No. of patients (%)** | |  | **No. of patients (%)** | |
| **Characteristic** | **Married-DSW**  **N=3043** | **Married-Married**  **N=17623** | **P** | **Married-DSW**  **N=3043** | **Married-Married**  **N=17623** |
| **Year of diagnosis** |  |  |  |  |  |
| 1992-1997 | 46(2) | 651(4) | <.001 | 83(3) | 612(3) |
| 1998-2003 | 341(11) | 2538(14) | <.001 | 457(15) | 2445(14) |
| 2004-2009 | 927(30) | 5707(32) | <.001 | 1013(33) | 5628(32) |
| 2010-2015 | 1729(57) | 8727(50) | <.001 | 1490(49) | 8938(51) |
| **Race** |  |  |  |  |  |
| White | 2483(82) | 14662(83) | <.001 | 2443(80) | 14667(83) |
| Black | 326(11) | 1249(7) | <.001 | 386(13) | 1249(7) |
| Other | 234(8) | 1712(10) | <.001 | 214(7) | 1707(10) |
| **Insurance** |  |  |  |  |  |
| Private insurance | 2334(77) | 14062(80) | <.001 | 2390(79) | 13960(79) |
| Insured/no specifics | 421(14) | 2383(14) | <.001 | 368(12) | 2460(14) |
| Any Medicaid | 261(9) | 1048(6) | <.001 | 238(8) | 1079(6) |
| Uninsured | 27(1) | 130(1) | <.001 | 47(2) | 124(1) |
| **Grade** |  |  |  |  |  |
| I | 696(23) | 4139(23) | 0.423 | 676(22) | 4125(23) |
| II | 1365(45) | 7681(44) | 0.423 | 1333(44) | 7706(44) |
| III | 982(32) | 5803(33) | 0.423 | 1034(34) | 5792(33) |
| **Histology** |  |  |  |  |  |
| IDC | 2158(71) | 12283(70) | 0.395 | 2149(71) | 12301(70) |
| ILC | 314(10) | 1910(11) | 0.395 | 282(9) | 1922(11) |
| Other | 571(19) | 3430(19) | 0.395 | 612(20) | 3400(19) |
| **AJCC T Stage** |  |  |  |  |  |
| pT1 | 2077(68) | 12923(73) | <.001 | 2183(72) | 12809(73) |
| pT2 | 633(21) | 3225(18) | <.001 | 594(20) | 3264(19) |
| pT3 | 108(4) | 509(3) | <.001 | 100(3) | 525(3) |
| pT4 | 92(3) | 403(2) | <.001 | 63(2) | 431(2) |
| Any T, Mets | 133(4) | 563(3) | <.001 | 104(3) | 594(3) |
| **AJCC N Stage** |  |  |  |  |  |
| pN0 | 2381(78) | 13757(78) | 0.309 | 2352(77) | 13770(78) |
| pN1 | 434(14) | 2621(15) | 0.309 | 451(15) | 2601(15) |
| pN2 | 124(4) | 611(3) | 0.309 | 141(5) | 605(3) |
| pN3 | 104(3) | 634(4) | 0.309 | 100(3) | 647(4) |
| **ER** |  |  |  |  |  |
| Negative | 643(21) | 3860(22) | 0.352 | 719(24) | 3846(22) |
| Positive | 2400(79) | 13763(78) | 0.352 | 2324(76) | 13777(78) |
| **PR** |  |  |  |  |  |
| Negative | 1078(35) | 6452(37) | 0.217 | 1148(38) | 6426(36) |
| Positive | 1965(65) | 11171(63) | 0.217 | 1895(62) | 11197(64) |
| **Surgery** |  |  |  |  |  |
| Nonsurgery | 179(6) | 666(4) | <.001 | 116(4) | 716(4) |
| BCS | 1168(38) | 6419(36) | <.001 | 1110(36) | 6475(37) |
| Mastectomy | 1696(56) | 10538(60) | <.001 | 1817(60) | 10432(59) |
| **Radiotherapy** |  |  |  |  |  |
| No | 2102(69) | 11955(68) | 0.183 | 2032(67) | 11982(68) |
| Yes | 941(31) | 5668(32) | 0.183 | 1011(33) | 5641(32) |
| **Chemotherapy** |  |  |  |  |  |
| No | 2280(75) | 11973(68) | <.001 | 2015(66) | 12145(69) |
| Yes | 763(25) | 5650(32) | <.001 | 1028(34) | 5478(31) |
| **Age (years)** |  |  |  |  |  |
| 20-40 | 43(1) | 385(2) | <.001 | 179(6) | 341(2) |
| 40-50 | 221(7) | 2469(14) | <.001 | 496(16) | 2254(13) |
| 50-65 | 768(25) | 7326(42) | <.001 | 938(31) | 7070(40) |
| ≥65 | 2011(66) | 7443(42) | <.001 | 1430(47) | 7958(45) |
