# Supplementary material for: Dynamic changes in marital status and survival in women with breast cancer: a population-based study
Source: Sci Rep. 2021 Mar 8;11:5421. doi: 10.1038/s41598-021-84996-y (PMC7940486; doi:10.1038/s41598-021-84996-y)
Supplement: Supplementary file 6 — Supplementary Table 6. [file 41598_2021_84996_MOESM6_ESM.docx]

Appendix table 6. Baseline demographic and tumor characteristics of patients according to marital status in SEER database.

|  | **Unmatched** | |  | **Matched** | |
| --- | --- | --- | --- | --- | --- |
|  | **No. of patients (%)** | |  | **No. of patients (%)** | |
| **Characteristic** | **DSW-Married**  **N=558** | **DSW-DSW**  **N=7333** | **P** | **DSW-Married**  **N=558** | **DSW-DSW**  **N=7333** |
| **Year of diagnosis** |  |  |  |  |  |
| 1992-1997 | 14(3) | 347(5) | 0.026 | 18(3) | 339(5) |
| 1998-2003 | 77(14) | 1167(16) | 0.026 | 98(18) | 1154(16) |
| 2004-2009 | 182(33) | 2400(33) | 0.026 | 182(33) | 2394(33) |
| 2010-2015 | 285(51) | 3419(47) | 0.026 | 260(47) | 3447(47) |
| **Race** |  |  |  |  |  |
| White | 439(79) | 5798(79) | 0.738 | 429(77) | 5797(79) |
| Black | 88(16) | 1086(15) | 0.738 | 96(17) | 1088(15) |
| Other | 31(6) | 449(6) | 0.738 | 33(6) | 448(6) |
| **Insurance** |  |  |  |  |  |
| Private insurance | 435(78) | 5283(72) | <.001 | 381(68) | 5320(73) |
| Insured/no specifics | 78(14) | 1143(16) | <.001 | 113(20) | 1120(15) |
| Any Medicaid | 38(7) | 867(12) | <.001 | 61(11) | 849(12) |
| Uninsured | 7(1) | 40(1) | <.001 | 2(0) | 44(1) |
| **Grade** |  |  |  |  |  |
| I | 132(24) | 1807(25) | 0.031 | 146(26) | 1794(24) |
| II | 229(41) | 3320(45) | 0.031 | 228(41) | 3315(45) |
| III | 197(35) | 2206(30) | 0.031 | 184(33) | 2225(31) |
| **Histology** |  |  |  |  |  |
| IDC | 403(72) | 4982(68) | 0.107 | 381(68) | 5003(68) |
| ILC | 55(10) | 806(11) | 0.107 | 57(10) | 803(11) |
| Other | 100(18) | 1545(21) | 0.107 | 120(22) | 1527(21) |
| **AJCC T Stage** |  |  |  |  |  |
| pT1 | 411(74) | 5208(71) | 0.64 | 400(72) | 5221(71) |
| pT2 | 101(18) | 1425(19) | 0.64 | 96(17) | 1420(19) |
| pT3 | 18(3) | 253(3) | 0.64 | 23(4) | 250(3) |
| pT4 | 11(2) | 208(3) | 0.64 | 16(3) | 204(3) |
| Any T, Mets | 17(3) | 239(3) | 0.64 | 22(4) | 238(3) |
| **AJCC N Stage** |  |  |  |  |  |
| pN0 | 438(78) | 5775(79) | 0.773 | 442(79) | 5770(79) |
| pN1 | 76(14) | 1046(14) | 0.773 | 72(13) | 1050(14) |
| pN2 | 25(4) | 268(4) | 0.773 | 24(4) | 270(4) |
| pN3 | 19(3) | 244(3) | 0.773 | 20(4) | 243(3) |
| **ER** |  |  |  |  |  |
| Negative | 114(20) | 1484(20) | 0.956 | 119(21) | 1484(20) |
| Positive | 444(80) | 5849(80) | 0.956 | 439(79) | 5849(80) |
| **PR** |  |  |  |  |  |
| Negative | 190(34) | 2528(34) | 0.875 | 196(35) | 2524(34) |
| Positive | 368(66) | 4805(66) | 0.875 | 362(65) | 4809(66) |
| **Surgery** |  |  |  |  |  |
| Nonsurgery | 28(5) | 378(5) | 0.882 | 31(6) | 376(5) |
| BCS | 224(40) | 3014(41) | 0.882 | 225(40) | 3011(41) |
| Mastectomy | 306(55) | 3941(54) | 0.882 | 302(54) | 3946(54) |
| **Radiotherapy** |  |  |  |  |  |
| No | 374(67) | 5153(70) | 0.117 | 386(69) | 5137(70) |
| Yes | 184(33) | 2180(30) | 0.117 | 172(31) | 2196(30) |
| **Chemotherapy** |  |  |  |  |  |
| No | 379(68) | 5770(79) | <.001 | 421(75) | 5712(78) |
| Yes | 179(32) | 1563(21) | <.001 | 137(25) | 1621(22) |
| **Age (years)** |  |  |  |  |  |
| 20-40 | 16(3) | 42(1) | <.001 | 4(1) | 57(1) |
| 40-50 | 62(11) | 366(5) | <.001 | 21(4) | 422(6) |
| 50-65 | 247(44) | 1700(23) | <.001 | 178(32) | 1759(24) |
| ≥65 | 233(42) | 5225(71) | <.001 | 354(63) | 5095(69) |
